# Impact of Surgical Margins on Overall Survival after Gastrectomy for Gastric Cancer: A Validation of Japanese Gastric Cancer Association Guidelines on a Western Series

**DOI:** 10.1245/s10434-021-11010-0

**Published:** 2022-01-01

**Authors:** Marianna Maspero, Carlo Sposito, Antonio Benedetti, Matteo Virdis, Maria Di Bartolomeo, Massimo Milione, Vincenzo Mazzaferro

**Affiliations:** 1 Upper GI and HPB Surgery, Fondazione IRCCS Istituto Nazionale Tumori, Milan, Italy; 2 Medical Oncology, Fondazione IRCCS Istituto Nazionale Tumori, Milan, Italy; 3 Pathology, Fondazione IRCCS Istituto Nazionale Tumori, Milan, Italy; 4 Department of Oncology and Hemato-Oncology, University of Milan, Milan, Italy

## Abstract

**Purpose:**

No consensus exists on the resection extent needed to ensure oncological safety in gastrectomy for gastric adenocarcinoma (GAC). This study aims to assess the impact of margin adequacy according to Japanese Gastric Cancer Association (JGCA) guidelines on overall survival (OS).

**Patients and Methods:**

Patients who underwent surgery for stage I–III GAC at our institution between 2010 and 2017 were included. Margin adequacy according to JGCA, National Comprehensive Cancer Network (NCCN), and European Society for Medical Oncology (ESMO) guidelines was assessed, and their predictive value on OS was evaluated with Harrell’s *C*-index. Patients were analyzed according to their margins’ adherence to JGCA guidelines, and a propensity score matching (PSM) was run. Indication to either total gastrectomy (TG) or distal gastrectomy (DG) according to each guideline was also assessed.

**Results:**

A total of 279 patients were included, of whom 220 (79%) underwent DG. Adequate margins according to JGCA were obtained in 209 patients (75%). On multivariate analysis, JGCA margin adequacy was independently associated with OS, together with American Society of Anesthesiologist class, neoadjuvant chemotherapy, lymphadenectomy extent, R0 resection, and postoperative N stage. After PSM, patients with JGCA adequate margins showed better OS, recurrence-free survival (RFS), and local RFS than patients with JGCA inadequate margins. For 220 DG, JGCA guidelines would have recommended TG in 25 patients (11%), NCCN in 30 (14%), and ESMO in 90 (41%) (*p* < 0.001).

**Conclusion:**

Adequacy of surgical resection margins to JGCA guidelines leads to improved survival outcomes and allows for a more organ-preserving approach than Western guidelines.

Gastric adenocarcinoma (GAC) is the fifth most frequently diagnosed cancer and the third leading cause of cancer-related death worldwide.^1^ Although chemotherapy and chemoradiation can improve outcomes in selected patients, surgical resection remains the only potentially curative treatment^2^ for GAC.

Several areas of controversy still exist on the proper extent of surgical resection during gastrectomy for GAC. There is currently no consensus on the adequate distance between the tumor and the resection margins to ensure a complete tumor excision and minimize the risk of local recurrence. Recently there has been an increase in the awareness that the extent of gastrectomy influences the quality of life^3,4^ and the nutritional status^5^ after surgery and that organ-preserving surgery should be performed whenever possible.

Recommendations on the extent of resection in the Guidelines of the major Eastern and Western cancer Societies are heterogeneous.

The National Comprehensive Cancer Network (NCCN) guidelines^6^ recommend resection margins > 4 cm from the gross tumor for resectable T1b–T3 tumors. T4 tumors require en bloc resection of the involved structures, while Tis or T1a tumors may be candidates for endoscopic resection.

In the European Society of Medical Oncology (ESMO) guidelines,^7^ the extent of resection is determined by the preoperative stage; endoscopic resection may be carried out for very early gastric cancers (T1a) if they are clearly confined to the mucosa, well differentiated, ≤ 2 cm, and non-ulcerated. For stage Ib–III GAC, distal gastrectomy (DG) may be carried out if a macroscopic proximal margin of 5 cm can be achieved between the tumor and the gastroesophageal junction for intestinal type cancers; for diffuse cancers, a margin of 8 cm is recommended. If those margins cannot be guaranteed, total gastrectomy (TG) should be performed

The Japanese Gastric Cancer Association (JGCA) guidelines^8^ recommend a margin > 2 cm for T1 tumors. For tumors ≥ T2, a proximal resection margin of at least 3 cm is recommended in case of expansive growth pattern, while an infiltrative growth pattern requires at least 5 cm. If this cannot be achieved, the proximal resection margin should be analyzed by frozen section.

Differently from the Western guidelines, the JGCA guidelines take into account the growth pattern of the tumor, thus allowing for a more patient-tailored and organ-sparing surgery while pursuing similar oncologic outcomes. With this in mind, starting from 2010, we adopted a surgical approach to gastrectomy for GAC aimed at adhering to JGCA guidelines whenever allowed by the intraoperative patient and tumor conditions.

This study analyzes the collected experience of patients undergoing gastrectomy for GAC, and assesses the impact of the adequacy to the resection margins recommended by JGCA guidelines on long-term outcomes. Secondarily, we aim to evaluate whether pursuing JGCA guidelines might result in more conservative gastric surgery with respect to Western guidelines.

## Patients and Methods

### Patient Selection and Study Endpoints

From a prospectively collected database, we identified a cohort of consecutive patients who underwent gastric resection for GAC at our institution between 1 January 2010 and 31 December 2017. Inclusion criteria were: age > 18 years, confirmed histology of GAC, and gastrectomy performed with curative intent. Exclusion criteria were: palliative surgery, R2 resection, esophagogastric resection, histological diagnosis different from adenocarcinoma, and stage IV disease.

Out of the 430 consecutive patients who underwent gastrectomy for gastric cancer during the study period, 151 patients were excluded: 13 for palliative surgery, 39 for esophagogastric resection, 62 for histology different from adenocarcinoma, 36 for stage IV disease, and 1 with macroscopic residual disease at transection margins (R2). Overall, the study cohort included 279 patients.

The study protocol followed the Declaration of Helsinki and its updates.^9^ Data analysis was approved by the institutional review board as a retrospective, observational study to be reported according to the STROBE^10^ guidelines (Strengthening the Reporting of Observational Studies in Epidemiology).

The primary endpoint of this study was to assess the impact of adequate resection margins according to JGCA guidelines on overall survival (OS) of patients undergoing gastrectomy for cancer. Secondary endpoints were: the impact of resection margins on recurrence-free survival (RFS) and local recurrence-free survival (LRFS), the predictive power of JGCA margins on OS with respect to NCCN and ESMO guidelines, and whether or not JCGA allowed a more conservative surgery with respect to other Western-based guidelines.

### Therapeutic Protocol

All patients underwent preoperative staging including performance status evaluation, physical examination, chest-abdominal contrast-enhanced computed tomography (ceCT), lab tests, and upper endoscopy with biopsy of the lesion to assess the histology according to the Lauren classification.^11^ In the case of suspected lymph node invasion or T stage > T2, the patients were investigated with upper endoscopic ultrasound and positron emission tomography. At the end of their preoperative work-up, all patients were staged according to the AJCC eight edition guidelines for gastric cancer.^12^

Patients received either distal gastrectomy (DG) or total gastrectomy (TG). Removal of a second tier of lymph nodes in the extraperigastric areas (D2 lymphadenectomy) was added whenever possible, with a minority of patients (Table 1) receiving only a D1 lymphadenectomy as a consequence of an estimated high risk of postoperative complications and low benefit, due to age and comorbidities, in the case of more extended lymphadenectomy. The extent of gastric resection was decided according to tumor size, tumor location, and intraoperative evaluation in order to guarantee adequate resection margins according to the JGCA classification. Intraoperative frozen section analysis of the proximal and distal margins was routinely performed: in the case of tumor involvement or high risk of R1, the resection was extended, when feasible, with the aim of complying with JGCA guidelines.

**Table 1:** Characteristics of study population

	*n* (%) or median (range) *n* = 279
*Baseline demographic and clinical characteristics*	
Sex	
Female	126 (45.2)
Age (years)	67 (20–89)
BMI (kg/m^2^)	24 (16.5–41)
ASA	
1 2 3	30 (10.8) 215 (77.1) 33 (11.8)
Clinical TNM stage	
I IIA IIB III	111 (39.8) 40 (14.3) 48 (17.2) 80 (28.7)
Preoperative histology	
Intestinal Diffuse	171 (61.3) 108 (38.7)
Neoadjuvant chemotherapy	
No Yes	216 (77.4) 63 (22.6)
*Postoperative pathological characteristics*	
Tumor location	
Greater curvature Lesser curvature	86 (30.8) 193 (69.2)
Tumor location	
Upper 1/3 Mid 1/3 Lower 1/3	20 (7.2) 121 (43.4) 138 (49.5)
Tumor size (cm)	4 (0.4–19)
PRM (cm)	5 (0.5–16)
DRM (cm)	4 (0.2–25)
Margins adequacy (JGCA)	
Adequate	209 (74.9)
Margins adequacy (NCCN)	
Adequate	189 (67.7)
Margins adequacy (ESMO)	
Adequate	108 (38.7)
Histology	
Intestinal Diffuse Indeterminate	153 (54.8) 68 (24.4) 55 (19.7)
Growth pattern	
Expansive Infiltrative	42 (15.1) 206 (73.8)
Total examined lymph nodes	33 (5–71)
Total positive lymph nodes	1 (0–45)
Lymph node sampling	
Adequate (≥ 16)	256 (91.8)
R0 resections	273 (97.8)
Postoperative T stage	
T0 T1 T2 T3 T4	3 (1,1) 101 (36.2) 32 (11.5) 96 (34.4) 47 (16.8)
Postoperative N stage	
N0 N1 N2 N3	114 (43.3) 60 (19.9) 48 (17.4) 57 (20.5)
Postoperative TNM stage	
0 I II III	3 (1.1) 100 (35.8) 79 (28.3) 97 (34.8)
Adjuvant chemotherapy	
No Yes	130 (46.6) 145 (52)

*BMI* body mass index, *ASA* American Society of Anesthesiologists, *PRM* proximal resection margin, *DRM* distal resection margin, *JGCA* Japanese Gastric Cancer Association, *NCCN* National Comprehensive Cancer Network, *ESMO* European Society of Medical Oncology

Postoperative follow-up consisted of complete lab tests and ceCT every 4 months for the first 2 years, and every 6 months thereafter. All patients received an upper endoscopy 1 year after surgery, then every 2 years. Neoadjuvant and/or adjuvant chemotherapy was administered according to tumor stage and current standards of care (see Results and Table 1).

### Data Collection and Terminology

The variables related to the type of surgery and the extent of the lymphadenectomy were defined according to the JGCA definitions.^8^ Clinical and pathological staging refer to the TNM-AJCC eighth edition Cancer Staging Manual.^12^ Adequacy of resection margins was assessed at pathology on a case-by-case basis, and defined according to the JGCA, NCCN, and ESMO recommendations.^6–8^ Tumor size was defined as the maximum diameter of the lesion. A lymph node sampling of at least 16 lymph nodes was considered adequate.^13^ Ninety-day postoperative complications were graded according to the Dindo–Clavien classification,^14^ and considered as “major” when graded > IIIa. Local recurrence was defined as an endoscopically detected and biopsy-proven tumor recurrence at the proximal margin.

### Organ-Sparing Assessment

The indication to either TG or DG according to each guideline was calculated in each individual patient, assuming a minimum distance of 4 cm from the cardias to perform DG for lesions located on the lesser curvature (LC) and of 8 cm for lesions located on the greater curvature (GC). If, after resection with adequate margins (i.e., tumor size + margins according to each guideline), this distance was not met, the portion of the stomach that would remain would not be sufficient to perform a DG. The length of the LC and GC were retrospectively approximated according to the formulas described by Lee et al.:^15^

- Greater curve = 17.47 + 0.02 × age + 0.06 × body weight;
- Lesser curve = 3.32 + 0.05 × age + 0.98 × sex + 0.05 × height + 0.03 × body weight

To calculate whether the minimum distance between the tumor and the surgical margins was met, we used the following formulas:

- For lesions in the lesser curve (LC) area: if [LC length − (tumor length + margins)] > 4 cm, then DG would have been indicated, otherwise TG
- For lesions in the greater curve (GC) area if [GC length − (tumor length + margins)] > 8 cm, then DG would have been indicated, otherwise TG

### Statistical Analysis

All categorical variables were reported as number of cases and percentages. Continuous variables were reported as mean ± standard deviation (SD) or as median and range, depending on the data distribution. Distribution of continuous variables was assessed with the Shapiro–Wilk’s test. Categorical variables were analyzed using the chi-squared test; continuous variables were analyzed with the Student’s *t*-test or Mann–Whitney test, as appropriate.

Overall survival (OS) was computed as the interval between the date of surgery and the date of death for any reason, with censoring at the date of last follow-up in alive patients. RFS was computed as the interval between surgery and the date on which tumor recurrence was recorded at any site, with censoring at the date of death or last follow-up in recurrence-free patients. LRFS was computed as the interval between surgery and the date on which local recurrence was recorded, with censoring at the date of death or last follow-up in recurrence-free patients. Proportional hazard assumption was verified by Schonfeld residual analysis, and survival curves were obtained with the Kaplan–Meier method and compared by means of log-rank test. Median follow-up time was calculated with the reverse Kaplan–Meier method. Cox regression analysis was performed to assess the variables independently associated with OS. Variables with *p* ≤ 0.05 on univariate analysis were included in the multivariable analysis. Since JGCA, NCCN, and ESMO guidelines share the covariate “proximal resection margin,” Harrell’s *C*-index was calculated to evaluate the discriminatory power on survival of each guideline. To avoid collinearity, only the one with the highest “*C*” was included in multivariable analysis. A Cox regression analysis assessing the association between time to recurrence (TTR) and margin adequacy according to the different guidelines was also carried out.

After grouping the patients according to their JGCA margin adequacy, the baseline characteristics of the two groups were compared. To overcome potential selection biases in patients with adequate and inadequate margins, a propensity score matching (PSM) analysis^16^ was conducted with a 1:2 nearest-neighbor matching and a caliper of 0.2. The PSM was run including variables with a well-known impact on survival and margin adequacy: tumor location (upper, middle, or lower third), type of gastrectomy (TG versus DG), nodal status, and TNM stage on pathology. After PSM, survival curves for OS, RFS, and LRFS were conducted by Kaplan–Meier methodology and analyzed using the Wilcoxon test. To overcome the potential confounding effect of pathological nodal status, we performed a subgroup analysis of the JGCA-IN and JGCA-OUT after-PSM cohorts, dividing them into pN0 and pN+ groups, and compared long-term outcomes between the pN0 JGCA-IN and JGCA-OUT cohorts, and between the pN+ JGCA-IN and JGCA-OUT cohorts.

In the organ-sparing surgery analysis, the differences in recommendations according to JGCA, NCCN, and ESMO were compared using one-way analysis of variance (ANOVA); *p* < 0.05 on ANOVA was further analyzed with Tukey’s test to assess significance between each guideline.

All analyses were two-sided, and statistical significance was defined as *p* < 0.05. Statistical analyses were performed with the IBM SPSS Advanced Statistics 24.0 package.

## Results

### Characteristics of the Population

The study includes 279 consecutive patients who underwent gastrectomy for GAC with curative intent. The main characteristics of the series are summarized in Table 1. DG was performed in 220 patients (78.9%), while 59 patients (21.1%) underwent TG. Intraoperative frozen sections were positive for distal margins tumor infiltration for six DG and no TG; of these, R0 was obtained in three by widening the duodenal resection. Frozen section was positive for proximal margins tumor infiltration in two DG and seven TG; of these, R0 was obtained in two by performing a TG and in four by widening the esophageal resection. Of the nine cases that resulted in R0 re-resections, five were adequate according to JGCA, four to NCCN, and zero to ESMO.

A D2 lymphadenectomy was associated in 240 patients (86%). The median length of hospital stay was 13 days (range 7–93 days), and the 90-day major morbidity and mortality rates were 12% (34 patients) and 2% (6 patients), respectively.

On pathology, R0 resections were confirmed in 273 (97.8%) cases. The median tumor size was 4 cm (range 0.4–19 cm). The median proximal resection margin (PRM) was 5 cm (range 0.5–16 cm), while the median distal resection margin was 4 cm (range 0.2–25 cm). Resection margins were adequate in 189 (67.7%) cases according to NCCN, in 108 (38.7%) cases according to ESMO, and in 209 (74.9%) cases according to JGCA guidelines. The lymph node sampling was adequate in 256 (91.8%) cases, with a median of 33 excised lymph nodes per patient (range 5–71). Clinical and pathological staging accounted for 111 (39.8%) and 100 (35.8%) stage I patients, 88 (31.5%) and 79 (28.3%) stage II patients, and 80 (28.7%) and 97 (34.8%) stage III patients, respectively. Overall concordance between clinical and pathological stage was 57% (160/279 patients). Of the 119 discordant cases, 70 (59%) were upstaged on pathology while 49 (41%) were downstaged.

### Survival Analysis

The median follow-up was 59.8 months [95% confidence interval (CI) 54.2–67.3 months]; during this period, 89 deaths and 64 recurrences (of which 8 were local recurrences) were registered. Of the eight local recurrences, one patient had received a R1 resection.

Out of 64 patients with a recurrence, 27 developed peritoneal carcinosis and 14 developed metastases in the liver, 14 in locoregional lymph nodes, 1 in retroperitoneal lymph nodes, 8 in the bone, 3 in the lung, and 3 in the ovary. Of the eight local recurrences, five occurred in the gastric remnant and three in the anastomosis; four had a concomitant recurrence in the locoregional lymph nodes, one in the lung, and one in the bone. Four had diffuse-type histology, while four had intestinal histology. Four had adequate margins according to JGCA, two according to NCCN, and none according to ESMO guidelines.

**Fig. 1:**
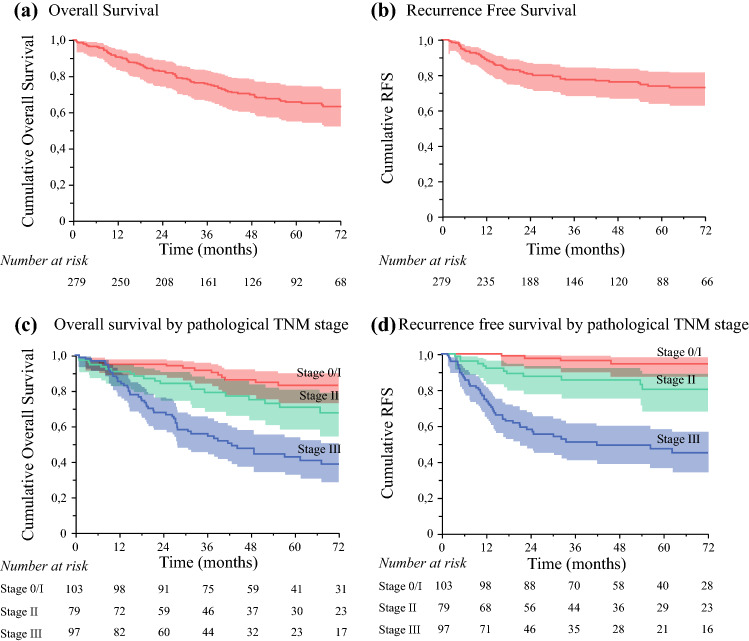
Survival curves in the overall study population according to pathological TNM stage. (a) Overall survival; (b) recurrence-free survival; (c) overall survival according to stage; (d) recurrence-free survival according to stage

The 1-, 3-, and 5-year OS of the entire series were 90.6%, 75.8%, and 65.5%, respectively (Fig. 1a), while the 1-, 3-, and 5-year RFS were 88.8%, 77.5%, and 74.1%, respectively (Fig. 1b). According to their pathology staging, the 5-year OS was 83.8%, 70.8%, and 42.9% for stage I, stage II, and stage III, respectively (Fig. 1c and Fig. 1d).

Univariate and multivariable analyses of OS are presented in Table 2. The ranking of the concordance Harrell’s *C*-index was 0.629 [standard error (SE) 0.023], 0.596 (SE 0.026), and 0.536 (SE 0.027) for JGCA, NCCN, and ESMO guidelines, respectively; therefore, only resection margins according to JGCA were analyzed in multivariable analysis. On multivariable analysis, American Society of Anesthesiologists (ASA) class (*p* < 0.0001), neoadjuvant chemotherapy (*p* = 0.002), lymphadenectomy extent (D2 versus D1: *p* = 0.01), R0 resection (*p* = 0.028), postoperative N stage (*p* < 0.0001), and margin adequacy according to JGCA (*p* = 0.0003) were independently associated with patients’ survival.

**Table 2:** Univariate and multivariate Cox regression analysis of factors independently associated with overall survival

	Univariate analysis			Multivariate analysis	
	3-Year OS (%)	5-Year OS (%)	*p*	HR (95% CI)	*p*
ASA classification					
1 2 3	75.9 80.6 40.8	64.3 70.8 30.6	**< 0.0001**	Ref. 4.5 (1.96–10.1)	**0.00003**
Neoadjuvant chemotherapy					
No Yes	81.4 54.9	70.4 47.8	**0.001**	Ref. 2.2 (1.36–3.58)	**0.002**
Gastrectomy extent					
Distal Total	80.5 57.7	70.7 45.8	**< 0.0001**		ns
Lymphadenectomy extent					
D1 D2	60 78.4	46.5 68.9	**0.006**	Ref. 0.35 (0.2–0.6)	**0.010**
Radicality on pathology					
R0 R1			**0.003**	Ref. 3.4 (1.2–10)	**0.028**
Margins adequacy (JGCA)					
Adequate Inadequate	82.7 55.5	73.2 43.3	**< 0.0001**	Ref. 2.35 (1.5–3.7)	**0.0003**
Margins adequacy (NCCN)					
Adequate Inadequate	81 65	72.8 51	**< 0.0001**		–
Margins adequacy (ESMO)					
Adequate Inadequate	78.9 73.9	71.3 62.1	0.110		
Tumor location					
Upper 1/3 Mid 1/3 Lower 1/3	62.2 76.9 76.9	42.7 66.1 68.4	**0.006**		ns
pT stage					
T1 T2 T3 T4	92.8 80.8 66.4 55.8	81.1 80.8 53.7 47.4	**< 0.0001**		ns
pN stage					
N0 N1 N2 N3a N3b	87.7 85.7 58.7 58.6 48.9	77.3 83.1 45.2 49,.9 19.6	**< 0.0001**	Ref. 0.93 (0.44–1.9) 2.2 (1.2–3.1) 3.4 (1.7–6.6) 4.8 (2.3–10.3)	**0.00003**
pTNM stage					
I II III	92.6 79.2 56	83.8 70.8 42.9	**< 0.0001**		–

*p* values < 0.05 are indicated in bold

*OS* overall survival, *95% CI* 95% confidence interval, *HR* hazard ratio, *ASA* American Society of Anesthesiology, *JGCA* Japanese Gastric Cancer Association, *NCCN* National Comprehensive Cancer Network, *ESMO* European Society of Medical Oncology

On univariate Cox regression analysis, the association of margin adequacy according to the various guidelines with TTR was as follows: for JGCA, hazard ratio (HR) 0.336 (95% CI 0.205–0.551, *p* < 0.0001); for NCCN, HR 0.47 (95% CI 0.288–0.769, *p* = 0.003); for ESMO, HR 0.43 (0.239–0.782, *p* = 0.006).

### Propensity Score Matching for JGCA Margin Adequacy

Considering the independent prognostic value of margin adequacy according to JGCA, patients were divided into two groups: JGCA-IN (with adequate margins, *n* = 209) and JGCA-OUT (with inadequate margins, *n* = 70). For the PSM, 105 JGCA-IN patients were matched with the 70 JGCA-OUT patients. Pre- and post-matching preoperative and intraoperative characteristics of the two groups are presented in Table 3: after PSM, pre- and intraoperative characteristics of the two cohorts were comparable.

**Table 3:** Characteristics of the JGCA-IN and JGCA-OUT cohorts before and after propensity score matching (PSM)

	Before PSM			After PSM		
	JGCA-IN (*n* = 209)	JGCA-OUT (*n* = 70)	*P*	JGCA-IN (*n* = 105)	JGCA-OUT (*n* = 70)	*p*
Age (years)	65 (11.9)	65 (12.6)	0.960	64 (12.3)	65 (12.6)	0.637
Sex						
Female Male	100 (47.9%) 109 (52.1%)	26 (37.1%) 44 (62.9%)	0.129	46 (43.8%) 59 (56.2%)	26 (37.1%) 44 (62.9%)	0.380
BMI	24.7 (4.5)	24.9 (3.8)	0.610	24.5 (4.3)	24.9 (3.8)	0.497
ASA classification						
1 2 3	21 (10%) 162 (77.5%) 26 (12.5%)	9 (13%) 53 (76.8%) 6 (8.7%)	0.717	13 (12.4%) 78 (74.3%) 14 (13.3%)	9 (13%) 53 (76.8%) 6 (8.7%)	0.503
cT stage						
T1 T2 T3 T4	30 (14.3%) 94 (45%) 72 (34.5%) 13 (6.2%)	2 (2.9%) 25 (35.7%) 35 (50%) 8 (11.4%)	**0.006**	7 (6.7%) 33 (31.4%) 53 (50.5%) 12 (11.4%)	2 (2.9%) 25 (35.7%) 35 (50%) 8 (11.4%)	0.701
cN stage						
N0 N+	129 (61.7%) 80 (38.3%)	30 (42.9%) 40 (57.1%)	**0.006**	53 (50.5%) 52 (49.5%)	30 (42.9%) 40 (57.1%)	0.323
Preoperative stage						
I IIA IIB III	95 (45.4%) 29 (13.9%) 34 (16.3%) 51 (24.4%)	16 (22.9%) 11 (15.7%) 14 (20%) 29 (41.4%)	**0.006**	29 (27.6%) 11 (10.5%) 24 (22.9%) 41 (39.5%)	16 (22.9%) 11 (15.7%) 14 (20%) 29 (41.4%)	0.681
CEA						
< 5 ng/ml > 5 ng/ml	163 (78%) 20 (22%)	45 (80.4%) 11 (19.6%)	0.089	80 (87%) 12 (13%)	45 (80.4%) 11 (19.6%)	0.282
Preoperative histology						
Intestinal Diffuse	130 (62.2%) 79 (37.8%)	41 (58.6%) 29 (41.4%)	0.589	53 (50.5%) 52 (49.5%)	41 (58.6%) 29 (41.4%)	0.293
Neoadjuvant chemotherapy	171 (81.8%) 38 (18.2%)	45 (64.3%) 25 (35.7%)	**0.005**	77 (73.3%) 28 (26.7%)	45 (64.3%) 25 (35.7%)	0.202
Gastrectomy extent						
Distal Total	176 (84.2%) 33 (15.8%)	44 (62.9%) 26 (37.1%)	**< 0.0001**	77 (73.3%) 28 (26.7%)	44 (62.9%) 26 (37.1%)	0.142
Lymphadenectomy extent						
D1 D2	29 (13.9%) 180 (85.1%)	10 (14.3%) 60 (85.7%)	0.932	11 (10.5%) 94 (90.5%)	10 (14.3%) 60 (85.7%)	0.247
PRM (cm)	6 (2–16)	2.5 (0.5–4)	**< 0.0001**	5.5 (2–16)	2.5 (0.5–4)	**< 0.001**
DRM (cm)	4 (0.2–21)	4 (0.5–25)	0.276	4 (0.2–19)	4 (0.5–25)	0.100
Lymph node sampling adequacy						
Adequate Inadequate	191 (91.4%) 18 (8.6%)	65 (92.9%) 5 (7.1%)	0.699	97 (92.4%) 8 (7.6%)	65 (92.9%) 5 (7.1%)	0.906
pN stage						
*pN0* *pN+*	98 (47%) 111 (53%)	16 (23%) 54 (77%)	**0.0004**	23 (22%) 82 (78%)	16 (23%) 54 (77%)	0.999

*p* values < 0.05 are indicated in bold

Data are presented as number (percentage), mean (standard deviation), or median (range), as appropriate. *BMI* body mass index, *ASA* American Society of Anesthesiologists, *cT,N,M* clinical T, N, M stage, *CEA* carcinoembryonic antigen, *PRM* proximal resection margin, *DRM* distal resection margin

**Fig. 2:**
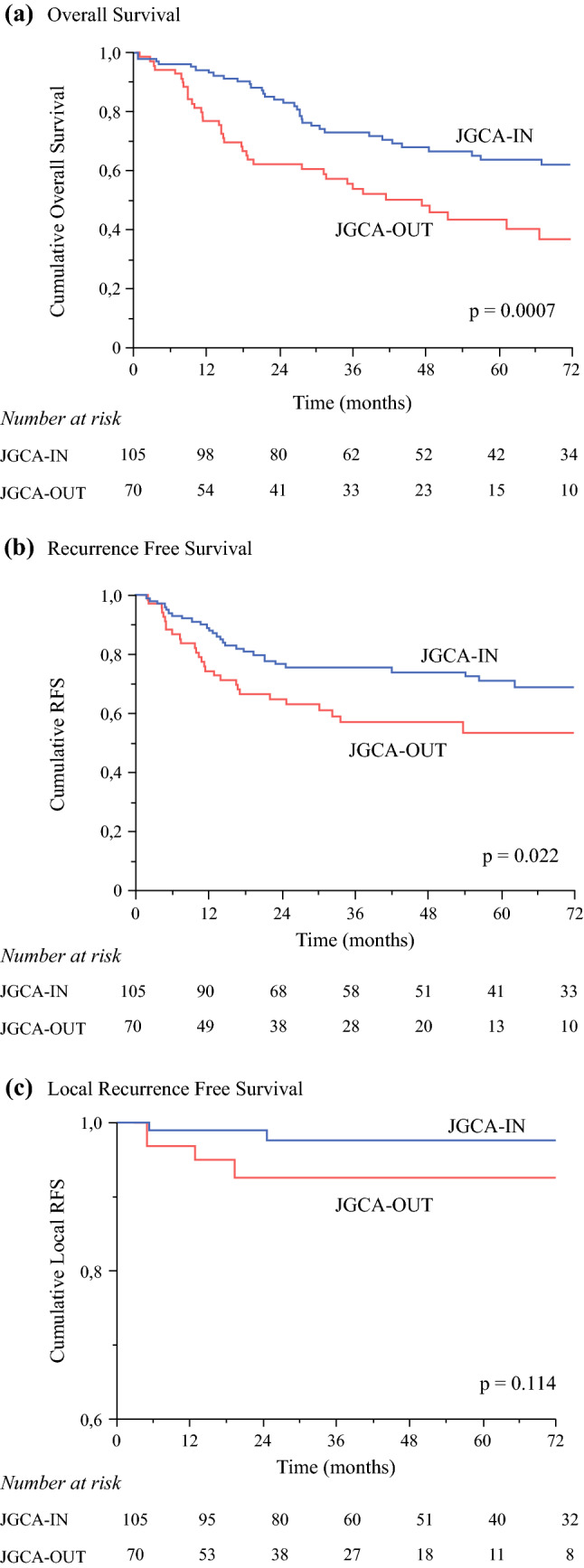
Survival curves in patients meeting or not meeting JGCA guidelines (JGCA-IN versus JGCA-OUT) after propensity score matching. (a) Cumulative overall survival; (b) cumulative recurrence-free survival; (c) cumulative local recurrence-free survival

The Kaplan–Meier survival curves for JGCA-IN and JGCA-OUT patients after PSM are shown in Fig. 2, where significant advantage in patient outcomes was detected for those operations meeting JGCA criteria. The 5-year OS, RFS, and LRFS for JGCA-IN versus JGCA-OUT were 64% versus 42% (*p* = 0.0007), 71% versus 53% (*p* = 0.022), and 98% versus 93% (*p* = 0.114), respectively.

The two post-matching cohorts were further divided into pN0 and pN+ patients. For pN0 patients, the 5-year OS, RFS, and LRFS for JGCA-IN versus JGCA-OUT were 90% versus 42% (*p* = 0.002), 96% versus 58% (*p* = 0.03), and 100% versus 93% (*p* = 0.199), respectively. For pN+ patients, the 5-year OS, RFS, and LRFS for JGCA-IN versus JGCA-OUT were 55% versus 39% (*p* = 0.02), 63% versus 51% (*p* = 0.119), and 97% versus 92% (*p* = 0.333), respectively.

### Organ-Sparing Surgery

**Fig. 3:**
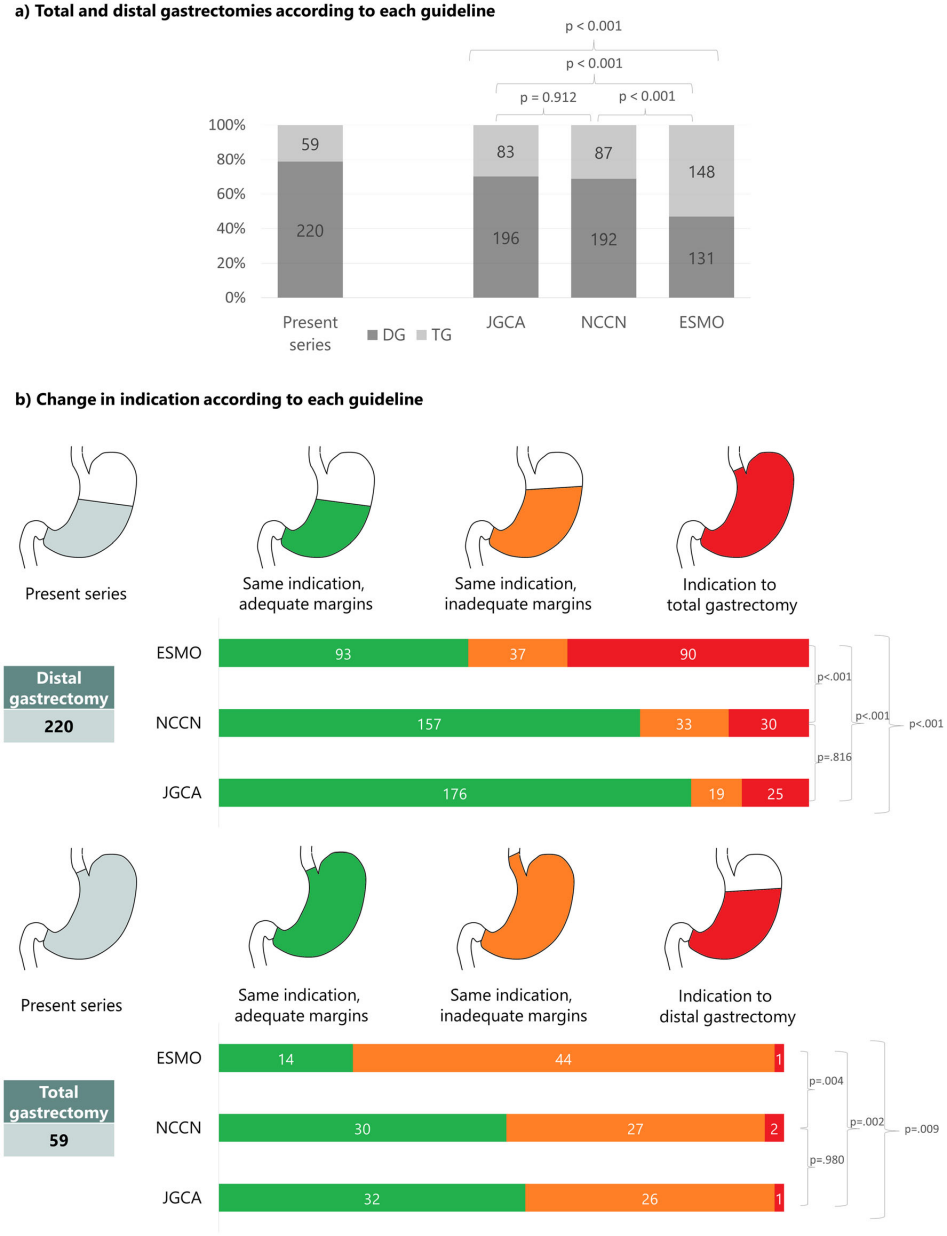
Observed indication to either total gastrectomy (TG) or distal gastrectomy (DG) and reclassification according to different guidelines. (a) The number of TG and DG that were actually performed (present series), and the number of TG and DG that would have been performed according to each guideline. (b) The indication that was actually given would have changed according to each guideline: the same indication with similar margins (i.e., same indication, adequate margins); the same indication, but with wider margins (i.e., same indication, inadequate margins); or a different indication (i.e., TG downstaged to DG, or DG upstaged to TG)

Figure 3a presents the distribution of the surgical strategy adopted in the present series compared with the recommendations of the reference guidelines. In 196/220 (70%) patients undergoing DG, such indication would have been confirmed by the JGCA guidelines. That percentage decreases to 69% (192 patients) for NCCN guidelines and to 47% (131 patients) for ESMO guidelines (*p* < 0.001).

Figure 3b shows how the DG and the TG that were actually performed would be reclassified according to each guideline. Of the 59 total gastrectomies performed, only one case according to JGCA, two according to NCCN, and one according to ESMO (*p* = 0.774) would be considered as overtreatments because a DG would have been indicated. Conversely, the number of cases in which the total gastrectomies performed would be considered as undertreatment because of inadequate margins was 26 (44%) according to JGCA, 27 (44%) according to NCCN, and 44 (74%) according to ESMO (*p* = 0.009). Finally, of the 220 DG performed, the number of patients that should have undergone a TG to comply with the margins length indicated by each guideline would have been 25 (11%) according to JGCA, 30 (14%) according to NCCN, and 90 (41%) according to ESMO (*p* < 0.001).

## Discussion

In our series of Western patients undergoing gastrectomy for adenocarcinoma, resection margin adequacy according to JGCA guidelines was associated with improved overall survival on multivariate analysis. Even after minimization of possible selection bias through PSM, patients meeting the JGCA guidelines achieved a better outcome (i.e., OS, RFS, and LRFS) compared with those patients receiving a gastrectomy beyond JGCA recommendations.

Incidentally, the short- and long-term outcomes observed in this study are comparable to the current literature;^17,18^ this speaks in favor of preservation of the expected outcome despite the adoption of an organ-sparing approach.

A definite conclusion on the prognostic impact of margins of resection in gastrectomies for cancer, especially when found microscopically positive or suboptimal in width, is far from being reached, as results of several studies on the topic have produced conflicting results.

The historical recommendation is to perform extended gastric resections with margins of at least 6 cm.^19^ In recent years, this dogma has been challenged, with some authors going as far as saying that efforts to achieve a certain resection distance should be abandoned, as they found no correlation between positive resection margins and prognosis.^20,21^ In contrast, other authors reported an association between positive margins and decreased OS in all stages of GAC.^22–25^ Others have found this association only in patients with lower T stages and node-negative diseases,^26–31^ possibly because in more advanced gastric cancers the prognosis is driven by the T and N involvement rather than by the resection margin.

In the presented study, R1 resections were independently associated with worsened survival, and one of the observed eight local recurrences occurred after R1 resection, thus suggesting that radical tumor-free margins (R0) should remain a goal of surgery for GAC.

Consensus on the adequate length of proximal resection margin (PRM) in the case of gastrectomy for GAC is equally lacking, as the evidence upon which major guidelines are based (i.e., JGCA, NCCN, and ESMO guidelines) is poorly discussed. Several reports^20,32–35^ conclude that PRM length has no impact on OS, while other large series^36^ recommend a PRM of 2.1–4.0 cm in solitary-type and 4.1–6.0 cm in infiltrative GAC.

The JGCA guidelines indicate an individualized approach to PRM according to tumor stage and morphology, which may result in a more organ-sparing resection for patients with early non-infiltrative tumors. In our center, we sought to follow such a tailored approach guided by preoperative staging and intraoperative evaluation of the specimen and of frozen sections of the PRM, aiming at more conservative, organ-sparing oriented gastrectomies for GAC. On final pathology, we found about 75% of the consecutively collected 279 patients in whom JCGA guidelines had been effectively adhered to. On multivariable analysis, adequate margins according to JGCA guidelines were independently associated with improved OS. JGCA margin adequacy showed better discriminatory power for survival with respect to margin compliance following Western-based guidelines, as demonstrated by the higher *C*-index of the former with respect to NCCN and ESMO indications. Indeed, while margin adequacy according to JGCA and NCCN guidelines was significantly associated with improved overall survival on univariate analysis, this was not the case for adequacy to ESMO guidelines. This shows that an aggressive, demolitive surgical approach, such as that recommended by ESMO guidelines, may not lead to improved oncological outcomes while potentially compromising morbidity and quality of life. Although margin adequacy to any of the guidelines showed an association with improved TTR on univariate Cox regression analysis, JGCA had the lowest HR and the narrower CI (HR 0.336, 95% CI 0.205–0.551, *p* < 0.0001).

To further evaluate the impact of JGCA recommendations on patients’ outcome, potential confounders were addressed. As JGCA-OUT patients were more likely to have a more advanced preoperative tumor stage with respect to JGCA-IN (T stage > T2 in 61.4% versus 40.7%; N+ in 57.1% versus 38.3%; preoperative stage > I in 76.1% versus 54.6%, respectively), leading to more frequent indication to TG (37.1% versus 15.8%), the achievement of adequate margins in that group might have been influenced by technical conditions related to the higher incidence of advanced tumors in unfavorable locations. In the case of inadequate distance from the PRM but with negative PRM on intraoperative frozen section analysis, the advanced preoperative stage and the intraoperative detection of macroscopically evident nodal disease may have prompted the decision not to perform a TG in the case of lesions located in the middle or lower part of the stomach and, similarly, not to extend the resection to the intrathoracic esophagus in the case of lesions located in the upper part of the stomach. To adjust the differences between patients meeting JGCA recommendations and those who did not, a PSM was applied; all JGCA-OUT patients found at least one match in the JGCA-IN group, and after PSM, the two groups became comparable. JGCA-IN patients maintained significantly better 5-year OS with respect to JGCA-OUT patients (64% versus 42%, *p* = 0.0007). Such improved survival could be partially explained by the improvement in RFS and LRFS observed when margins adhered to JGCA guidelines; indeed, although LRFS never reached significance, this could be due to the low number of patients who experienced a local recurrence, since an improved trend could be appreciated in JGCA-IN patients. These findings validate and further support the use of JGCA recommendations also in Western patients.

When the post-matching cohorts were divided according to their pathological nodal status, both the pN0 and pN+ JGCA-IN cohorts maintained significantly better OS than the respective JGCA-OUT cohorts. However, the impact was more pronounced in pN0 patients (5-year OS of 90% in the JGCA-IN patients versus 42% in the JGCA-OUT) than in pN+ patients (5-year OS 55% in the JGCA-IN patients versus 39% in the JGCA-OUT). These findings are in line with those studies^26,30,31^ suggesting that positive nodal status is an important driver of the prognosis in GAC and indicating that margin adequacy is especially relevant in pN0 patients.

Together with tumor-related outcomes, two other important aspects should be considered when planning a gastrectomy for GAC: postoperative morbidity and quality of life. Morbidity has been shown to be related to the resection extent, with TG yielding a higher rate of postoperative complications than DG,^37–40^ especially when associated with esophagectomy.^17^ Total gastrectomy has also been associated with worsened quality of life^3,4,41–43^ and higher rate of dysphagia.^43^ For these reasons, an organ-sparing approach should be adopted whenever compatible to tumor stage and when technically feasible.

In our study on a series of Western patients, JGCA guidelines allowed more organ-sparing procedures than NCCN and ESMO (Fig. 3a), with a lower number of TG compared with Western guidelines (30% versus 31% in NCCN and 47% in ESMO, *p* < 0.001) without compromising patient outcomes. When reclassifying the indication to gastrectomy, JGCA guidelines yielded the lowest number of TG with inadequate margins, i.e., those in which the resection should have been extended to the intrathoracic esophagus (44% versus 46% in NCCN and 75% in ESMO). Accordingly, the implementation of JGCA guidelines in Western patients could lead to better short- and long-term outcomes, thanks to the higher number of organ-sparing procedures.

Our study has several limitations. It is a retrospective, single-center observational study that takes into account a period of 8 years, during which changes in the clinical management of GAC and in surgical technique may have occurred, influencing our results. While PSM was used to minimize confounders, the presence of residual confounders cannot be excluded. In addition, indications according to each guideline were calculated with inferred margins, obtained through calculations based on anthropometric data, which may differ from in vivo/ex vivo actual measurements. Finally, the analysis was limited to the main guidelines, not considering the many nuances proposed in other surgical/oncology societies.

## Conclusions

The presented consecutive series of Western patients with GAC suggests that the adequacy of surgical resection margins according to JGCA guidelines leads to improved patient outcomes (i.e., OS, RFS, and LRFS). JGCA guidelines also allow for a more organ-sparing approach than NCCN and ESMO guidelines.

## References

[1] Bray F, Ferlay J, Soerjomataram I, Siegel RL, Torre LA, Jemal A (2018). Global cancer statistics 2018: GLOBOCAN estimates of incidence and mortality worldwide for 36 cancers in 185 countries. CA Cancer J Clin..

[2] Waddell T, Verheij M, Allum W (2014). Gastric cancer: ESMO–ESSO–ESTRO clinical practice guidelines for diagnosis, treatment and follow-up. Eur J Surg Oncol..

[3] Brenkman HJF, Tegels JJW, Ruurda JP (2018). Factors influencing health-related quality of life after gastrectomy for cancer. Gastric Cancer..

[4] Takahashi M, Terashima M, Kawahira H (2017). Quality of life after total vs distal gastrectomy with Roux-en-y reconstruction: use of the Postgastrectomy Syndrome Assessment Scale-45. World J Gastroenterol..

[5] Rosania R, Chiapponi C, Malfertheiner P, Venerito M (2016). Nutrition in patients with gastric cancer: an update. Gastrointest Tumors..

[6] National Comprehensive Cancer Network. Gastric Cancer. Version 2019. Accessed December 15, 2020. https://www.nccn.org/professionals/physician_gls/pdf/gastric.pdf.

[7] Smyth EC, Verheij M, Allum W, Cunningham D, Cervantes A, Arnold D (2016). Gastric cancer: ESMO Clinical Practice Guidelines for diagnosis, treatment and follow-up. Ann Oncol..

[8] Japanese Gastric Cancer Association (2017). Japanese gastric cancer treatment guidelines 2014 (ver. 4). Gastric Cancer..

[9] Association WM (2013). World medical association declaration of Helsinki: ethical principles for medical research involving human subjects. JAMA..

[10] von Elm E, Altman DG, Egger M, Pocock SJ, Gøtzsche PC, Vandenbroucke JP (2007). Strengthening the reporting of observational studies in epidemiology (STROBE) statement: guidelines for reporting observational studies. BMJ..

[11] Lauren P (1965). The two histological main types of gastric carcinoma: diffuse and so-called intestinal-type carcinoma. An attempt at a histo-clinical classification. Acta Pathol Microbiol Scand..

[12] Amin MB, Edge SB, Greene FL, Brierley JD (2017). AJCC cancer staging manual.

[13] Hu B, El Hajj N, Sittler S, Lammert N, Barnes R, Meloni-Ehrig A (2012). Gastric cancer: classification, histology and application of molecular pathology. J Gastrointest Oncol..

[14] Dindo D, Demartines N, Clavien P-A (2004). Classification of surgical complications: a new proposal with evaluation in a cohort of 6336 patients and results of a survey. Ann Surg..

[15] Lee EG, Kim TH, Huh YJ (2016). Anthropometric study of the stomach. J Gastric Cancer..

[16] Austin PC (2011). An introduction to propensity score methods for reducing the effects of confounding in observational studies. Multivariate Behav Res..

[17] Papenfuss WA, Kukar M, Oxenberg J (2014). Morbidity and mortality associated with gastrectomy for gastric cancer. Ann Surg Oncol..

[18] Wang J, Sun Y, Bertagnolli MM (2015). Comparison of gastric cancer survival between Caucasian and Asian patients treated in the United States: results from the Surveillance Epidemiology and End Results (SEER) database. Ann Surg Oncol..

[19] Bozzetti F, Bonfanti G, Bufalino R (1982). Adequacy of margins of resection in gastrectomy for cancer. Ann Surg..

[20] Postlewait LM, Squires MH, Kooby DA (2015). The importance of the proximal resection margin distance for proximal gastric adenocarcinoma: a multi-institutional study of the US Gastric Cancer Collaborative. J Surg Oncol..

[21] Squires MH, Kooby DA, Pawlik TM (2014). Utility of the proximal margin frozen section for resection of gastric adenocarcinoma: a 7-institution study of the US Gastric Cancer Collaborative. Ann Surg Oncol..

[22] Woo JW, Ryu KW, Park JY (2014). Prognostic impact of microscopic tumor involved resection margin in advanced gastric cancer patients after gastric resection. World J Surg..

[23] Nagata T, Ichikawa D, Komatsu S (2011). Prognostic impact of microscopic positive margin in gastric cancer patients. J Surg Oncol..

[24] Zhao B, Lu H, Bao S (2020). Impact of proximal resection margin involvement on survival outcome in patients with proximal gastric cancer. J Clin Pathol..

[25] Bissolati M, Desio M, Rosa F (2017). Risk factor analysis for involvement of resection margins in gastric and esophagogastric junction cancer: an Italian multicenter study. Gastric Cancer..

[26] Byoung CC, Hei CJ, Hye JC (2007). Prognostic impact of resection margin involvement after extended (D2/D3) gastrectomy for advanced gastric cancer: a 15-year experience at a single Institute. J Surg Oncol..

[27] Luo J, Jiang Y, Chen X (2020). Prognostic value and nomograms of proximal margin distance in gastric cancer with radical distal gastrectomy. Chin J Cancer Res..

[28] Sun Z, Li DM, Wang ZN (2009). Prognostic significance of microscopic positive margins for gastric cancer patients with potentially curative resection. Ann Surg Oncol..

[29] Falagas ME, Kasiakou SK (2005). Mesh-related infections after hernia repair surgery. Clin Microbiol Infect..

[30] Aurello P, Magistri P, Nigri G (2014). Surgical management of microscopic positive resection margin after gastrectomy for gastric cancer: a systematic review of gastric R1 management. Anticancer Res..

[31] Kim SH, Karpeh MS, Klimstra DS (1999). Effect of microscopic resection line disease on gastric cancer survival. J Gastrointest Surg..

[32] Lee CM, Jee YS, Lee JH (2014). Length of negative resection margin does not affect local recurrence and survival in the patients with gastric cancer. World J Gastroenterol..

[33] Kim A, Kim BS, Yook JH, Kim BS (2020). Optimal proximal resection margin distance for gastrectomy in advanced gastric cancer. World J Gastroenterol..

[34] Kim BS, Kim BS, Oh ST, Yook JH, Kim HS, Lee IS (2014). Appropriate gastrectomy resection margins for early gastric carcinoma. J Surg Oncol..

[35] Berlth F, Kim WH, Choi JH (2020). Prognostic impact of frozen section investigation and extent of proximal safety margin in gastric cancer resection. Ann Surg..

[36] Wang J, Liu J, Zhang G, Kong D (2017). Individualized proximal margin correlates with outcomes in gastric cancers with radical gastrectomy. Tumor Biol..

[37] Li Z, Bai B, Xie F, Zhao Q (2018). Distal versus total gastrectomy for middle and lower-third gastric cancer: a systematic review and meta-analysis. Int J Surg..

[38] Gockel I, Pietzka S, Gönner U, Hommel G, Junginger T (2005). Subtotal or total gastrectomy for gastric cancer: Impact of the surgical procedure on morbidity and prognosis—analysis of a 10-year experience. Langenbeck’s Arch Surg..

[39] Ji X, Yan Y, De BuZ (2017). The optimal extent of gastrectomy for middle-third gastric cancer: distal subtotal gastrectomy is superior to total gastrectomy in short-term effect without sacrificing long-term survival. BMC Cancer..

[40] Durán Giménez-Rico H, Diéguez Aguirre L, Ríos Pérez L (2020). Comparative study between total and subtotal gastrectomy for distal gastric cancer: meta-analysis of prospective and retrospective studies. Cir Esp..

[41] Rausei S, Mangano A, Galli F (2013). Quality of life after gastrectomy for cancer evaluated via the EORTC QLQ-C30 and QLQ-STO22 questionnaires: surgical considerations from the analysis of 103 patients. Int J Surg..

[42] Wu CW, Chiou JM, Ko FS (2008). Quality of life after curative gastrectomy for gastric cancer in a randomised controlled trial. Br J Cancer..

[43] Goh YM, Gillespie C, Couper G, Paterson-Brown S (2015). Quality of life after total and subtotal gastrectomy for gastric carcinoma. Surgeon..

